# Vapor‐Deposited Cs_2_AgBiCl_6_ Double Perovskite Films toward Highly Selective and Stable Ultraviolet Photodetector

**DOI:** 10.1002/advs.201903662

**Published:** 2020-04-22

**Authors:** Ming Wang, Peng Zeng, Zenghui Wang, Mingzhen Liu

**Affiliations:** ^1^ School of Materials and Energy University of Electronic Science and Technology of China Chengdu 611731 P. R. China; ^2^ Center for Applied Chemistry University of Electronic Science and Technology of China Chengdu 611731 P. R. China; ^3^ Institute of Fundamental and Frontier Sciences University of Electronic Science and Technology of China Chengdu 611731 P. R. China

**Keywords:** double perovskite films, selective detection, sequential vapor deposition, ultraviolet photodetectors

## Abstract

Double perovskites have shown great potentials in addressing the toxicity and instability issues of lead halide perovskites toward practical applications. However, fabrication of high‐quality double perovskite thin films has remained challenging. Here, sequential vapor deposition is used to fabricate high‐quality Cs_2_AgBiCl_6_ perovskite films with balanced stoichiometry, superior morphology, and highly oriented crystallinity, with an indirect bandgap of 2.41 eV. Using a diode structure, self‐powered Cs_2_AgBiCl_6_ ultraviolet (UV) photodetectors are demonstrated with high selectivity centered at 370 nm, with low dark current density (≈10^−7^ mA cm^−2^), high photoresponsivity (≈10 mA W^−1^), and detectivity (≈10^12^ Jones). Its detectivity is among the highest in all double‐perovskite‐based photodetectors reported to date and surpassing the performance of other perovskite photodetectors as well as metal oxide in the UV range. The devices also show excellent environmental and irradiation stability comparable to state‐of‐the‐art UV detectors. The findings help pave the way toward application of double perovskites in optoelectronic devices.

## Graphical abstract

Lead‐free Cs_2_AgBiCl_6_ double perovskite films with balanced stoichiometry, compact morphology, and high crystallinity are fabricated by a sequential vapor deposition method. Their application is explored as an ultraviolet photodetector which realizes low dark current density (≈10^−7^ mA cm^−2^), high detectivity (≈10^12 ^Jones), and stability (4 months). This work reveals the potential of Cs_2_AgBiCl_6_ double perovskites in ultraviolet detection.

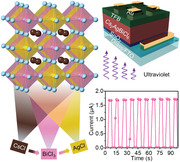

Photodetectors that can capture light to convert into electrical signals are critical to many industrial and military applications. Light‐absorbing material that converts incident photons to charge carriers is the key part of a photodetector. While a variety of conventional semiconductor materials has been successfully used, such as silicon (Si), group II–VI and III–V compounds, the expensive initial fabrication cost and mechanical inflexibility have hindered their broader application in scenarios such as low‐cost disposable devices, fast‐prototyping printable electronics, and flexible devices.

Recently, hybrid organic–inorganic perovskites have demonstrated fast and promising progresses toward realizing low‐cost, high performance flexible devices, largely thanks to their facile fabrication processes (via evaporation or solution), compatibility with flexible substrates, and excellent optoelectronic performance (efficiency of perovskite solar cells has exceeded 25% in just a few years).^[^ ^1^ ^]^ In general, typical hybrid halide perovskites employ the crystal structure of ABX_3_, where A, B, and X adopt organic cations (e.g., CH_3_NH_3_^+^), metal cations (Pb^2+^), and halide anions (I^−^, Br^−^, Cl^−^ or their mixed), respectively.^[^ ^2^ ^]^ With their long carrier diffusion length,^[^ ^3^ ^]^ high defect tolerance,^[^ ^4^ ^]^ long carrier lifetime, and high absorption coefficient,^[^ ^5^ ^]^ perovskite materials have enabled a plethora of optoelectronic devices, such as light emitting diodes,^[^ ^6^ ^]^ lasers,^[^ ^7^ ^]^ transistors,^[^ ^8^ ^]^ as well as photodetectors in ultraviolet, visible, and near infrared wavelengths.^[^ ^9^ ^]^ Despite all these remarkable successes, many perovskite‐based optoelectronics suffer from the intrinsic instability of the organic components and toxicity of Pb^2+^, two key compositions in the most commonly used halide‐based organic–inorganic hybrid perovskites. This has largely restricted their practical application.^[^ ^10^ ^]^ There have been research efforts in addressing these challenges,^[^ ^11^ ^]^ such as replacing the Pb^2+^ cation with Sn^2+^. However, the easy oxidation of Sn^2+^ to Sn^4+^ due to the high‐energy‐lying 5s orbitals leads to extremely low stability and poor charge transport properties in ambient conditions. To date, all the reported lead‐free perovskite photodetectors show slow response time of milliseconds and poor detectable light intensity of ≈µW cm^−2^, much inferior to their lead‐based counterparts.^[^ ^9a^ ^]^

Double perovskites, A_2_B(I)B’(III)X_6_, with two different halide cations B(I) and B’(III) substituting the toxic Pb^2+^, have been proposed as new alternatives to resolve the instability and the toxicity of the hybrid perovskites.^[^ ^12^ ^]^ For example, Cs_2_AgInCl_6_ has been reported with high photoluminescence efficiency and long‐term stability, which shows little photoluminescence decay after 1000 h at 150 °C.^[^ ^13^ ^]^ Besides, a number of studies show that photodetectors based on the double perovskite Cs_2_AgBiX_6_ (X = Cl, Br, and I) is a newly popular category for lead‐free double perovskite with indirect bandgaps. In particular, Cs_2_AgBiBr_6_ shows an indirect bandgap as seen from their optical measurements and electronic structure calculations.^[^ ^12^, ^14^ ^]^ A number of studies have demonstrated the Cs_2_AgBiBr_6_ double perovskites in the application of photodetectors with high‐sensitivity and fast‐response.^[^ ^15^ ^]^ Recently, Cs_2_AgBiBr_6_ perovskite in the form of single crystals has been demonstrated for high performance X‐ray detectors with low detection limit.^[^ ^16^ ^]^ Substituting Br with Cl is expected to further enhance detection application in the UV range as a result of the increased bandgap and smaller carrier effective mass (thus faster carrier diffusion).^[^ ^12^ ^]^ However, Cs_2_AgBiCl_6_ thin film materials and devices have remained elusive. The fabrication of Cs_2_AgBiCl_6_ materials so far is limited to nanocrystals^[^ ^17^ ^]^ that restricts further investigation of its optical properties as well as its application in optoelectronic devices.

In this work, we demonstrate high‐performance Cs_2_AgBiCl_6_ perovskite photodetector enabled by sequential vapor deposition technique.^[^ ^1^, ^18^ ^]^ By carefully tuning the compositional ratio and annealing process, we achieve Cs_2_AgBiCl_6_ films with balanced stoichiometry, high crystallinity, and superior morphology. Furthermore, we experimentally verify the indirect transition with large stokes shift in the Cs_2_AgBiCl_6_ double perovskites with an indirect bandgap of 2.41 eV. The planar‐type UV‐photodetectors are then designed and fabricated based on the vapor‐deposited Cs_2_AgBiCl_6_ films, showing high selectivity centered at 370 nm with a full width at half‐maximum (FWHM) of only 67 nm. The device exhibits excellent performance with low dark current density (≈10^−7^ mA cm^−2^), high photoresponsivity (≈10 mA W^−1^) and detectivity (≈10^12^ Jones), and excellent environment‐ and photo‐stability, which compare favorably with most UV photodetectors such as TiO_2_, SnO_2_, and GaN reported in literature.^[^ ^19^ ^]^

We use sequential vapor deposition^[^ ^18b^ ^]^ to fabricate Cs_2_AgBiCl_6_ double perovskite films (**Figure** **1**a). CsCl, BiCl_3_, and AgCl are deposited on the prepared substrates layer by layer, with the ratio of each layer carefully controlled to maintain the stoichiometry (see the Supporting Information for details). One complete cycle of sequential deposition of “CsCl‐BiCl_3_‐AgCl” results in a film thickness about 110 nm according to the cross‐section scanning electron microscopy (SEM) image (Figure 1d) and films with greater thicknesses can be obtained by simply repeating the deposition cycles. The double perovskite films are then annealed in nitrogen environment to fully crystallize. X‐ray photoelectron spectroscopy (XPS) measurements (Figure 1b) confirm the existence of Cs, Ag, Bi, and Cl in the perovskite film, where the XPS scans of peak details further indicate that the resultant Cs_2_AgBiCl_6_ double perovskite film has ideal stoichiometry (Figure S1, Supporting Information, and Figure 1c).

**Figure 1:**
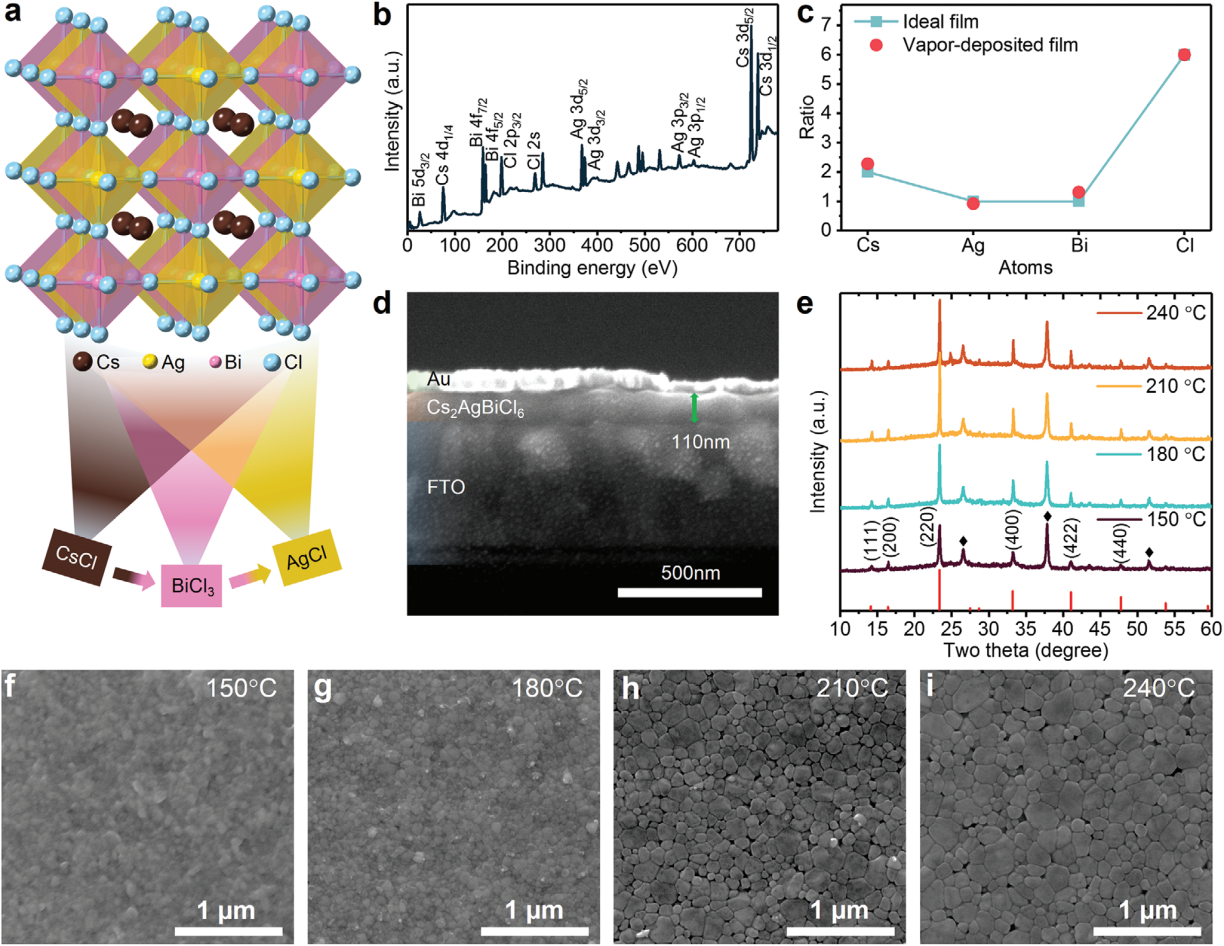
a) Scheme of sequential vapor deposition process and crystal structure of Cs_2_AgBiCl_6_ double perovskite. b) Wide‐range XPS scan of Cs_2_AgBiCl_6_ films deposited on fluorine doped tin oxide (FTO). c) The stoichiometry of vapor‐deposited films according to detailed XPS scanning in Figure S1 in the Supporting Information. d) Cross‐sectional SEM image of deposited films via one complete deposition cycle. e) XRD patterns of Cs_2_AgBiCl_6_ films annealed at different temperatures (the red line is standard diffraction patterns got from Inorganic Crystal Structure Database (ICSD) Coll. Codes 239874 and the diffraction peaks of FTO are labeled with diamond). SEM images of films annealed at f) 150 °C, g) 180 °C, h) 210 °C, and i) 240 °C.

X‐ray diffraction (XRD) patterns of the perovskite films annealed at different temperatures are consistent with standard diffraction patterns as well as values of single crystals^[^ ^12a^ ^]^ and nanocrystals in literature.^[^ ^17b,c^ ^]^ This confirms the formation of phase‐pure Cs_2_AgBiCl_6_ double perovskites from the sequential vapor deposition method (Figure 1e).^[^ ^20^ ^]^ It is found that the (220):(400) peak intensity ratio of our films is higher than the standard one, indicating a (220)‐preferred orientation (Figure S2, Supporting Information). In addition, the annealing temperature plays an important role in obtaining high quality films. The intensity and FWHM of main diffraction peak at 23.37° of (220) plane initially improve with temperature before deteriorate at elevated temperature (Figure S3b, Supporting Information) and the information about (400) plane is provided in Figure S3 in the Supporting Information. SEM images show that the film morphology exhibits similar temperature dependence: the film quality initially enhances until pinholes start to appear at higher annealing temperatures (Figure 1f–i). Based on the observed crystallinity and morphology at different annealing temperatures, we determine the optimal condition of 180 °C, which produces highly crystallized and pinhole‐free Cs_2_AgBiCl_6_ double perovskite films.

We also characterize the optical properties of the high‐quality Cs_2_AgBiCl_6_ perovskite film through its absorption and emission spectra (**Figure** **2**a). The perovskite exhibits weak absorption from 600 to 420 nm, followed by a sharp absorption peak between 420 and 350 nm. Similar absorption feature has been observed in other double perovskites such as Cs_2_AgBiBr_6_^[^ ^17^, ^21^ ^]^ and Cs_2_NaBiCl_6_^[^ ^17c^ ^]^ and is attributed to excitonic absorption^[^ ^17^, ^21^ ^]^ or direct bismuth s–p transition.^[^ ^17a^ ^]^ Under 370 nm excitation (from Xenon lamp with monochromator), the double perovskite film deposited on glass substrate exhibit strong photoluminescence (PL) (Figure 2a inset), confirming the high quality of the vapor‐deposited Cs_2_AgBiCl_6_ film, with an emission peak at 600 nm (Figure 2). The large shift between the absorption and emission peaks strongly suggests an indirect bandgap in the double perovskite material.^[^ ^22^ ^]^ We then fit the absorption data to an indirect transition in Tauc plot (Figure 2b). The intercepts of the first two linear regions at 2.08 and 2.76 eV correspond to phonon‐assisted light emissions by absorbing and emitting a phonon, respectively. This gives an indirect bandgap as 2.41 eV, consistent with calculated theoretical value^[^ ^12a^ ^]^ and experimental measurements with Cs_2_AgBiCl_6_ powders.^[^ ^12b^ ^]^ We further determine valence band minimum (VBM) and conduction band maximum (CBM) of the material using ultraviolet photoelectron spectroscopy (UPS). We measure a binding energy of 1.05 eV and cutoff energy of 16.27 eV (Figure 2c), from which we estimate the VBM and CBM of Cs_2_AgBiCl_6_ to be −6.00 and −3.59 eV, respectively (Figure 2d). This matches well with many commonly used electron transport layer (ETL, e.g., SnO_2_, TiO_2_) and hole transport layer (HTL, e.g., poly[bis(4‐phenyl) (2,4,6‐trimethylphenyl)amine] (PTAA), poly(3‐hexylthiophene‐2,5‐diyl) (P3HT), 2,2',7,7'‐tetrakis‐(N,N‐di‐4‐methoxyphenylamino)‐9,9'‐spirobifluorene (Spiro‐OMeTAD), poly[(9,9‐dioctylfluorenyl‐2,7‐diyl)‐co‐(4,4'‐(N‐(4‐sec‐butylphenyl)diphenylamine)] (TFB)) materials, suggesting that our double perovskite thin‐film material is promising for realizing high‐performance optoelectronic devices.

**Figure 2:**
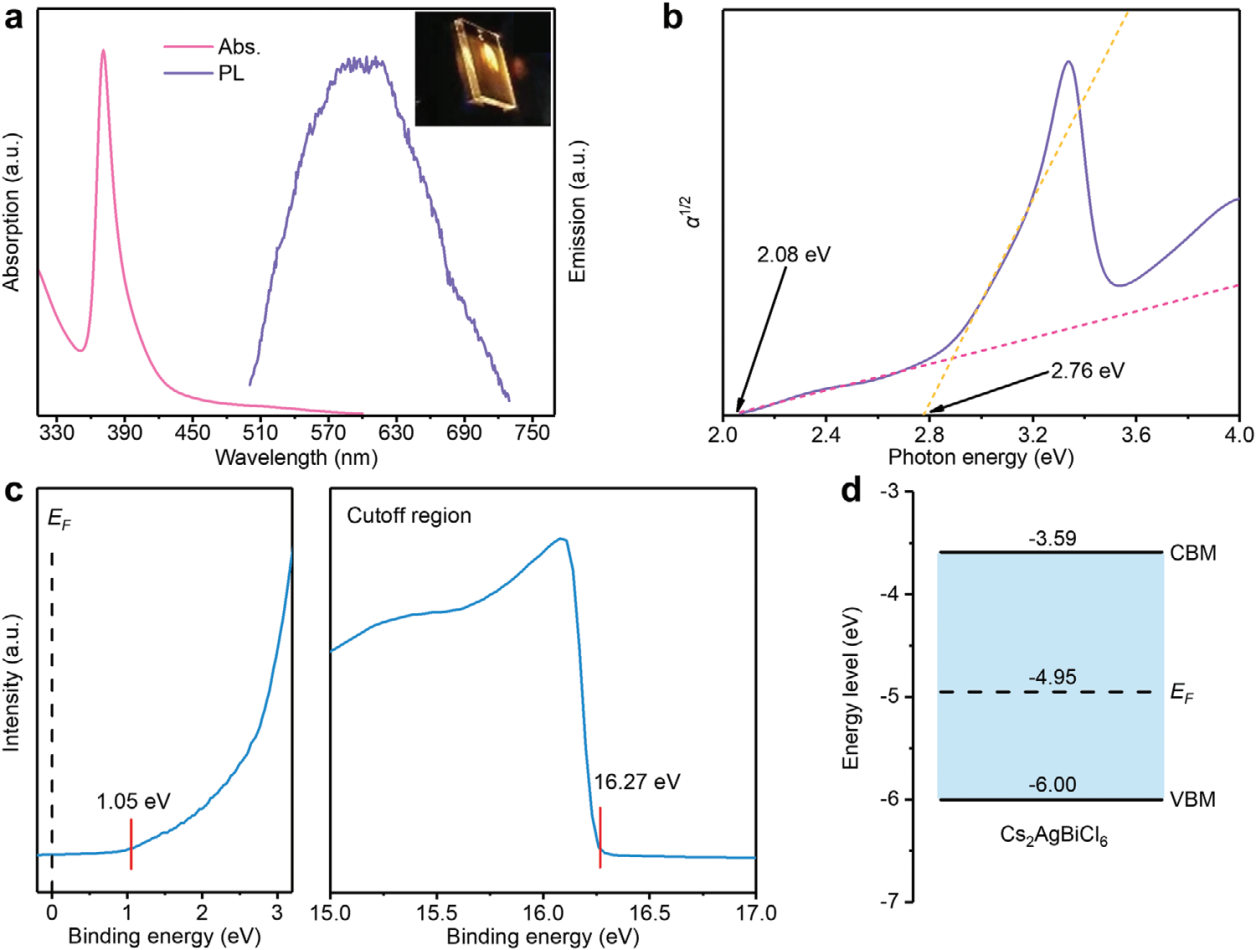
Optoelectronic characterization of Cs_2_AgBiCl_6_ films. a) Absorption and emission spectra of Cs_2_AgBiCl_6_ films (inset optical image shows the photoluminescence of film under 370 nm excitation). b) Indirect Tauc plot from absorption spectrum. c) UPS spectrum and d) energy level of Cs_2_AgBiCl_6_ double perovskite films.

To demonstrate the UV light sensing potential of the Cs_2_AgBiCl_6_ film, we fabricate UV photodetector using the double perovskite. The device configuration of FTO/ETL/perovskite/HTL/Au is employed for optimizing the UV photodetector performance. By examining different ETL/HTL combinations for effective charge transfer, we find that the combination of SnO_2_ and TFB produces the best device performance in terms of both photocurrent and dark current (Figure S4, Supporting Information).^[^ ^15b^ ^]^ We also vary the double perovskite film thickness (Figure S5a–c, Supporting Information) and determine that the device with 184 nm film thickness produces the highest ratio of photocurrent to dark current (Figure S6 and Table S1, Supporting Information) as well as most stable photoresponse over repeated illumination (Figure S7, Supporting Information). Thus, we use these optimized parameters for further evaluating the performance of Cs_2_AgBiCl_6_ UV photodetectors.

The spectral photoresponsivity (*R*) is a key figure‐of‐merit for photodetectors. The wavelength‐dependent *R* can be derived using the equation as^[^ ^15^, ^23^ ^]^

(1) $$\begin{array}{c} R=\mathrm{EQE}\frac{q}{h\upsilon } \end{array}$$

where EQE is the external quantum efficiency (Figure S8a, Supporting Information), *q* is the unit charge, *h* is the Planck constant, and υ is the wave frequency. The device exhibits good photoresponsivity between 438 and 300 nm in the UV‐A range, successfully rejecting most of visible light (**Figure** **3**b). Quantitatively, the narrow responsivity peak centered at 370 nm has an FWHM of only 67 nm, highlighting the excellent wavelength‐selectivity in UV‐A region of our Cs_2_AgBiCl_6_ photodetector.

**Figure 3:**
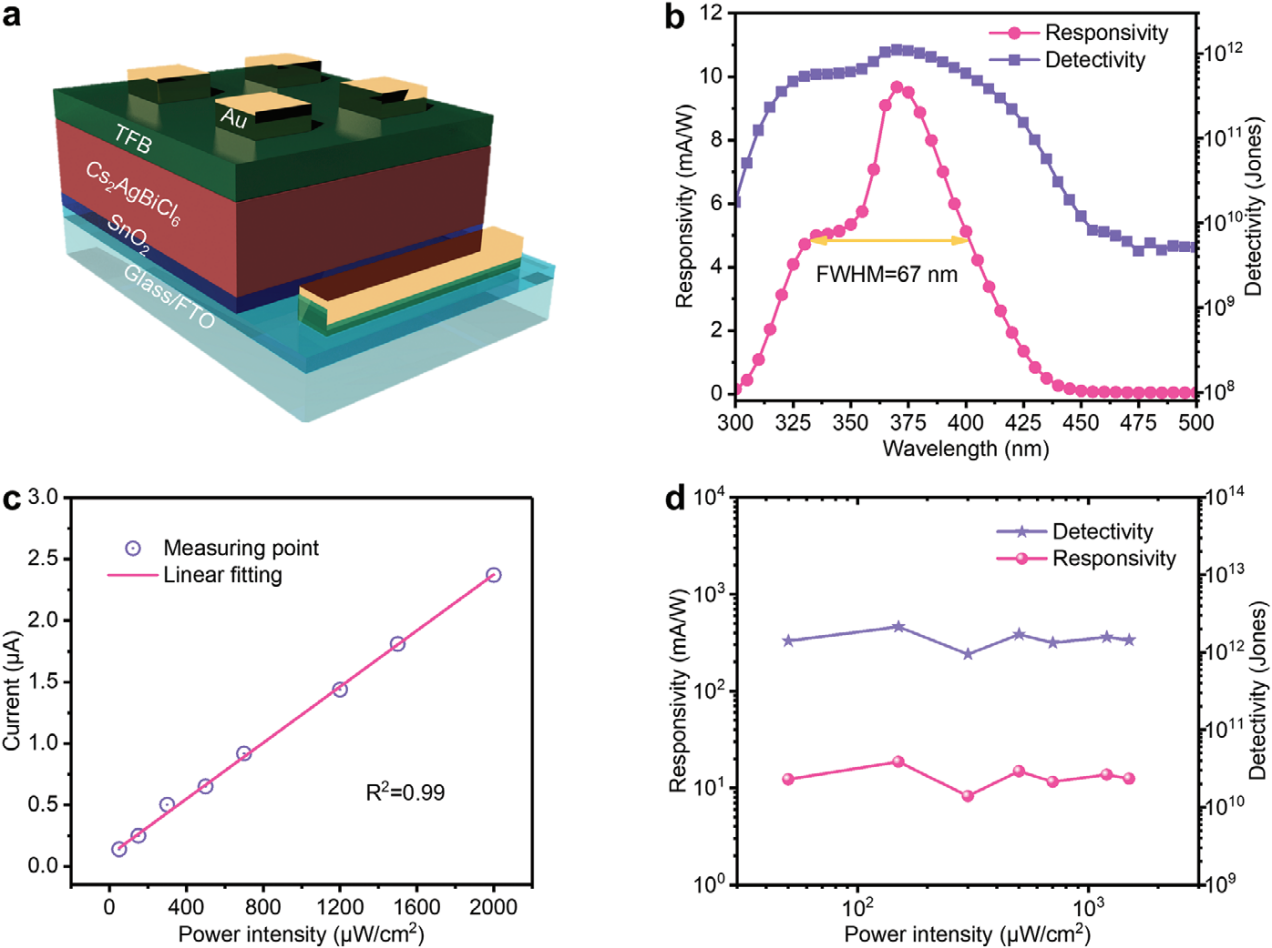
a) Device structure of Cs_2_AgBiCl_6_ ultraviolet photodetector. b) Wavelength‐dependent responsivity and detectivity of devices. c) Power‐dependent current under 0 V bias at light wavelength of 365 nm. d) Power intensity dependent responsivity and detectivity of double perovskite UV photodetectors.

Another important metric for photodetector is the detectivity D* which reflects the ability to detect weak signal. When the dark current is dominated by shot noise, it can be correlated with the responsivity through the equation as^[^ ^24^ ^]^

(2) $$\begin{array}{c} D^{*}=\frac{R}{{\left(2qJ_{d}\right)}^{1/2}}\; \end{array}$$

where *R* is the responsivity, *q* is the unit charge, and *J*_d_ is the dark current density. For the device we measure a very low *J*_d_ of 2.39 × 10^−7^ mA cm^−2^ (Figure S6 and Table S1, Supporting Information), which gives a maximum detectivity of 1.11 × 10^12^ Jones using the peak responsivity of 9.68 mA W^−1^ at 370 nm. We note that this value is two magnitudes higher than the other lead‐based perovskite UV photodetectors,^[^ ^25^ ^]^ which again demonstrates the unique advantage of Cs_2_AgBiCl_6_ films in the application of UV detection.

In order to evaluate the device performance under different incident light power, we measure the power‐dependent photocurrent under zero bias. By plotting the photocurrent as a function of light intensity, it shows a linear relationship from 50 to 2000 µW cm^−2^ (Figure 3c), suggesting that there is no energy barrier for carrier collection. Within the linear region, the Cs_2_AgBiCl_6_ works as a power‐sensitive photodetector which can also function as an effective UV light power meter. To further evaluate the linearity of the device, we calculate a power‐dependent responsivity from the slope of the curve between any two adjacent data points^[^ ^26^ ^]^ in Figure 3c, and the results are plotted in Figure 3d together with the power‐dependent detectivity. We find that both *R* and D* remain largely constant over almost two decades of power intensity, with values in excellent agreement with what is derived from EQE measurement. Even under light intensity as high as 1500 µW cm^−2^, the detectivity of the device maintains above 10^12^ Jones. Encouragingly, its detectivity is among the highest in all double‐perovskite‐based photodetectors reported to date, including those made of Cs_2_AgInCl_6_ and Cs_2_AgBiBr_6_ double perovskites (Table S2, Supporting Information),^[^ ^15^ ^]^ and surpassing the performance of other perovskite photodetectors^[^ ^25a^ ^]^ as well as metal oxide^[^ ^19b^ ^]^ in the UV range.

We further evaluate stability of the Cs_2_AgBiCl_6_ double perovskite material and devices toward endured operations. **Figure** **4**a shows the dynamic current–time (*I*–*t*) photoresponse of the photodetector under the repetitive and periodical illumination of 365 nm UV light. The ON and OFF states remain highly stable as the reversible and rapid switch from illumination to dark for 105 s with a very low baseline (i.e., dark current), confirming good stability and reversibility of the device. Moreover, the double perovskite material and device exhibit good environmental stability. Figure 4b shows the XRD spectra of a Cs_2_AgBiCl_6_ film exposed to air with 40% relative humidity (RH) without encapsulation for over 200 days. The crystallinity exhibits no deterioration throughout the extended period, but instead some slight increase in peak intensity. This has been observed in other perovskite films and was attributed to moisture‐assisted crystallization effects.^[^ ^27^ ^]^ At device level, a Cs_2_AgBiCl_6_ UV photodetector is stored in dry box (15% RH) at room temperature for over 120 d (Figure 4c), and the device performance shows no degradation.

**Figure 4:**
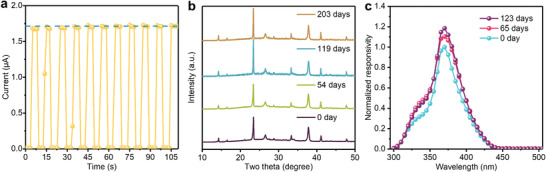
Stability of double perovskite UV photodetector. a) Continuous on–off photoresponse of Cs_2_AgBiCl_6_ UV photodetectors under 365 nm illumination at light power intensity of 1 mW cm^−2^. b) XRD patterns of fresh and aged Cs_2_AgBiCl_6_ films (in ambient environment with about 40% RH). c) Normalized responsivity of fresh and aged Cs_2_AgBiCl_6_ UV photodetectors (in dry box with about 15% RH).

In summary, we have successfully produced high‐quality Cs_2_AgBiCl_6_ double perovskite thin films via sequential vapor deposition method. We experimentally determine that the Cs_2_AgBiCl_6_ double perovskite thin films possess an indirect bandgap of 2.41 eV with large Stokes shift. By designing the device configuration, we further demonstrate planar‐type UV photodetectors based on the Cs_2_AgBiCl_6_ double perovskites, which exhibit high UV light selectivity centered at 370 nm with a responsivity peak width of 67 nm, a low dark current density of ≈10^−7^ mA cm^−2^, a high detectivity of ≈10^12 ^Jones, as well as good environmental and irradiation stability. It is worth noting that it is the first time to realize the self‐powered UV photodetectors based on Cs_2_AgBiCl_6_ double perovskite thin films through vapor deposition. The detectivity is among the highest in all double‐perovskite‐based photodetectors reported to date and surpassing the performance of other perovskite photodetectors as well as metal oxide in the UV range. Our work demonstrates that Cs_2_AgBiCl_6_ double perovskite devices hold great potential in applications.

## Experimental Section

##### Materials

BiCl_3_ (99.999%, Sigma‐Aldrich), CsCl (99.999%, Sigma‐Aldrich), AgCl (99.99%, Sigma‐Aldrich), PTAA (Xi'an Polymer Light Technology), Spiro‐OMeTAD (99.8%, Borun New Material Technology), P3HT (Xi'an Polymer Light Technology), TFB (Xi'an Polymer Light Technology), bis(trifluoromethane)sulfonimide lithium salt (Li‐TFSI) (99.95%, Sigma‐Aldrich), 4‐tert‐butylpyridine (tBP, 96%, Sigma‐Aldrich), and 1,2‐dichlorobenzene (Sigma‐Aldrich) were all used as received without further purification. The SnO_2_ colloidal solution (15% in H_2_O) was purchased from Alfa Aesar and was dispersed into deionized water as volume ratio of 1:3.

##### Preparation of Cs_2_AgBiCl_6_ Films

The sequential vapor deposition was carried out in vacuum environment under pressure below 1.0 × 10^−3^ Pa. After the optimization of deposition order, first CsCl, then BiCl_3_, and lastly AgCl were deposited layer by layer. According to the stoichiometric molar ratio, the thickness ration of each layer was calculated to be “CsCl: BiCl_3_: AgCl = 3.28: 2.57: 1.” One deposition cycle parameter of “CsCl‐98 nm, BiCl3‐77 nm, AgCl‐30 nm” was set. The films with larger thickness can be obtained by simply repeating the deposition cycle. After vapor deposition, the films were annealed in nitrogen‐filled glove box at 180 degrees centigrade (optimized) for 5 min.

##### Device Fabrication

The FTO substrate was cleaned with deionized water, acetone and ethanol successively in ultrasonic cleaner. After 10 min plasma treatment, tin oxide colloidal (diluted in deionized water with three‐fold volume) was casted on the FTO at 3000 rpm for 30 s. Then the substrate was annealed at 150 °C for 30 min in air to crystallize better. Subsequently, by sequential vapor deposition and annealing treatment, Cs_2_AgBiCl_6_ double perovskite films were formed on compact SnO_2_ layer. The TFB HTL solution was prepared according to ref. [14b]. After stirring, the filtered solution was spin coated on the top of Cs_2_AgBiCl_6_ films at 3000 rpm for 30 s in N_2_ filled glove box. Finally, the Gold electrode was deposited with the thickness about 100 nm by thermal evaporation under the pressure below 1 × 10^−3^ Pa.

##### Characterization

Bruker D8 Advance diffractometer with Cu *K*_*α*_ radiation (*λ* = 1.5418 Å) was used to do XRD test. Hitachi S‐4300 field‐emission electron microscope was used to do SEM characterizations, with electron energy of 10 keV. The UV–vis spectra were gained by using Carry 500 (Agilent Technologies). Steady‐state PL was conducted by using FluoTime 300 (PicoQuant). Ultraviolet photoelectron spectra (UPS) was measured with Thermo Fisher Scientific Escalab 250Xi system by using a He I discharge lamp (21.22 eV), and a bias voltage of −5 V was applied. X‐ray photoelectron spectroscopy (XPS) was performed on the same system as UPS. The EQE spectrum was measured with QE‐R (enlitechnology). The Keithley 4200 series digital source‐meter unit was used to measure the *J–V* curves of devices which were placed under 365 nm UV light source. The effective area of one photodetector is 0.09 cm^2^.

## Conflict of Interest

The authors declare no conflict of interest.

## Supporting information

Supporting Information
