# Supplementary material for: Vapor‐Deposited Cs2AgBiCl6 Double Perovskite Films toward Highly Selective and Stable Ultraviolet Photodetector
Source: Adv Sci (Weinh). 2020 Apr 22;7(11):1903662. doi: 10.1002/advs.201903662 (PMC7284202; doi:10.1002/advs.201903662)
Supplement: Supplementary file 1 — Supporting Information [file ADVS-7-1903662-s001.pdf]

Supporting Information

Vapor-deposited $Cs_2AgBiCl_6$ double perovskite films toward highly selective and stable ultraviolet photodetector

*Ming Wang, Peng Zeng, Zenghui Wang, and Mingzhen Liu**

M. Wang, Prof. P. Zeng, Prof. M. Liu
School of Materials and Energy, University of Electronic Science and Technology of China, Chengdu 611731, P. R. China
E-mail: mingzhen.liu@uestc.edu.cn

M. Wang, Prof. P. Zeng, Prof. M. Liu
Center for Applied Chemistry, University of Electronic Science and Technology of China, Chengdu 611731, P. R. China
E-mail: mingzhen.liu@uestc.edu.cn

Prof. Z. Wang
Institute of Fundamental and Frontier Sciences, University of Electronic Science and Technology of China, Chengdu 611731, P. R. China.

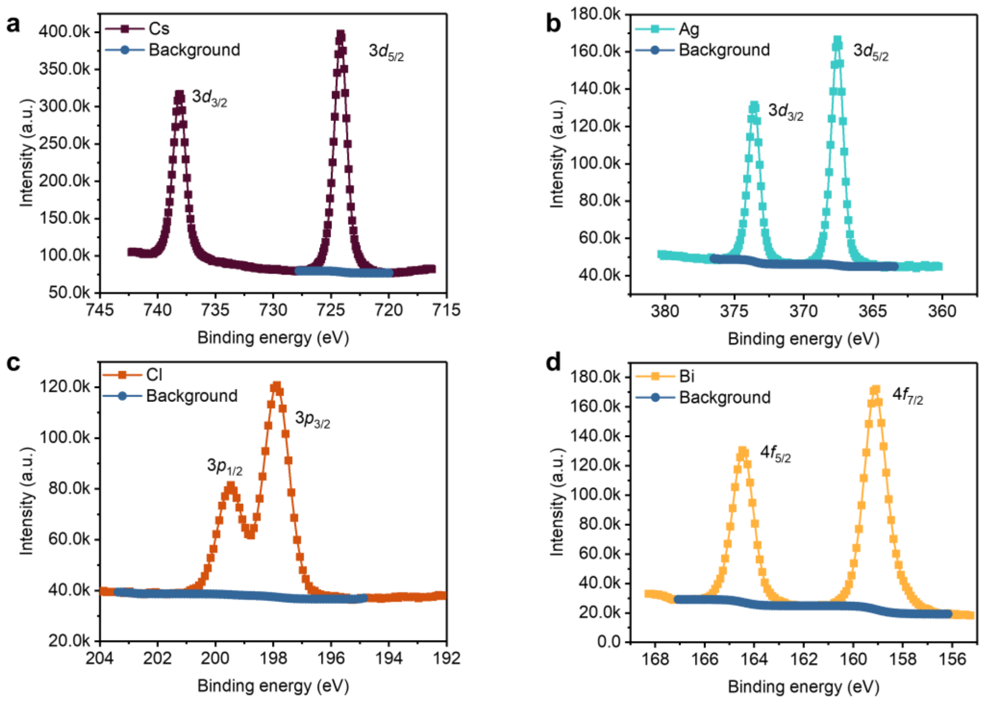

Figure S1. The detailed XPS scanning of (a) Cs 3d, (b) Ag 3d, (c) Cl 3p and (d) Bi 4f.

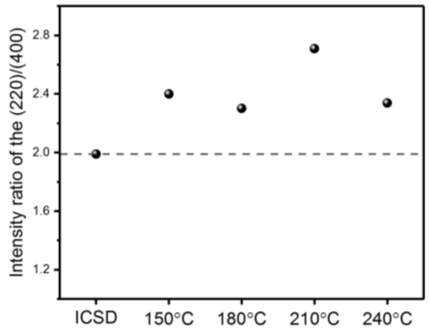

Figure S2. Comparison of the relative intensity ratios of the (220):(400) between the standard ICSD Coll. Codes 239874 and the obtained diffractions.

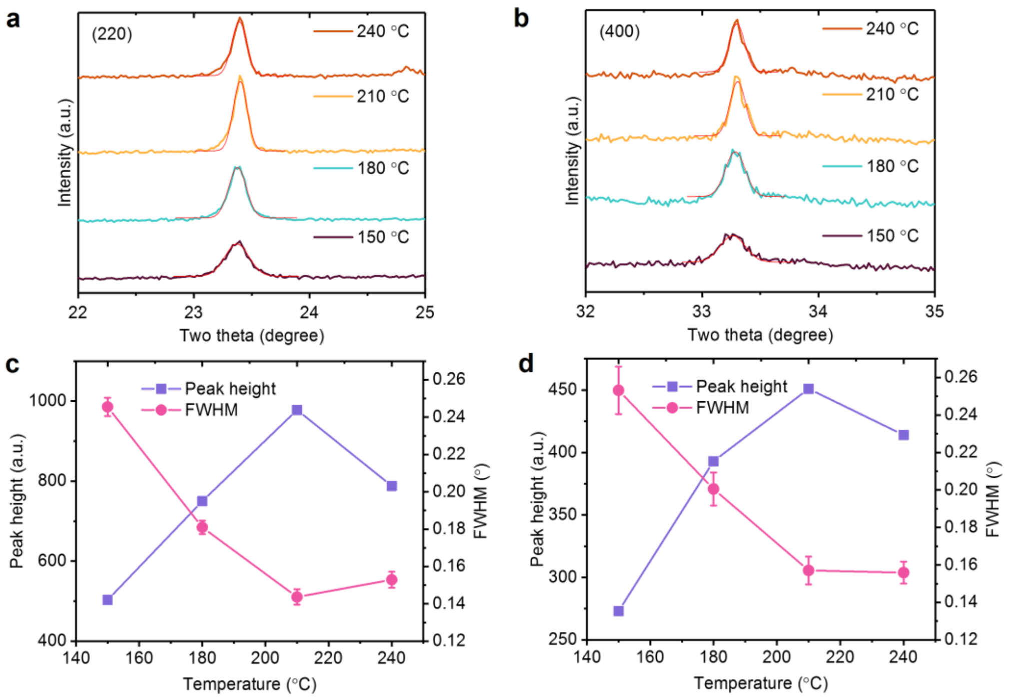

Figure S3. X-ray diffraction of (a) (220) and (b) (400) crystal face of $Cs_2AgBiCl_6$ double perovskite films annealed at different temperature for 5 minutes (the thin red lines are fitted Gaussian curves). (c) and (d) show annealing-temperature-dependent peak height and FWHM of (220) and (400), respectively. Error bars for FWHM indicate errors of the Gaussian fitting.

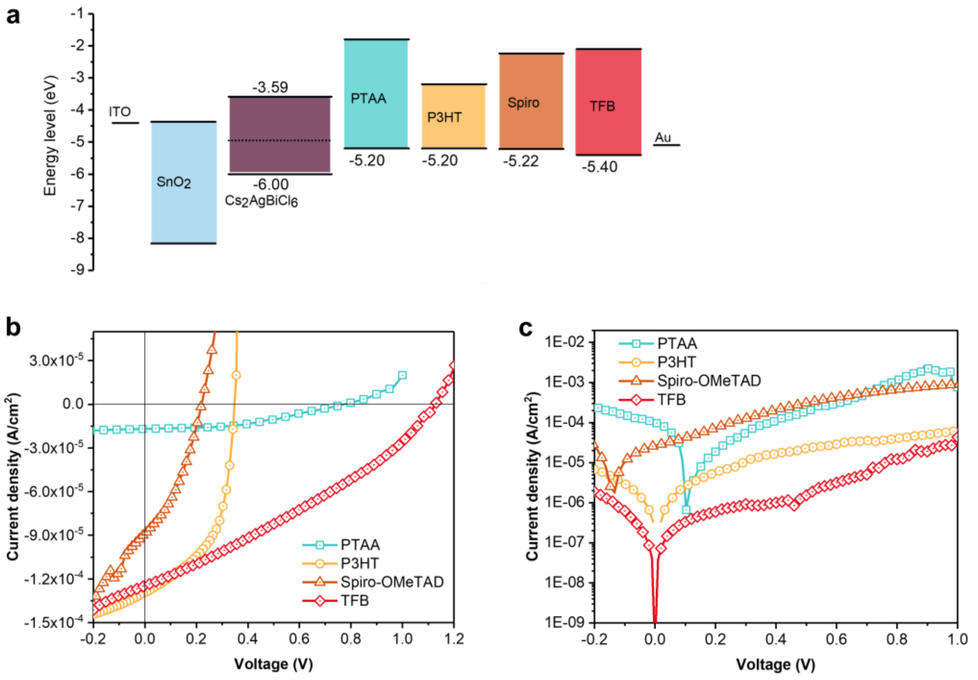

Figure S4. (a) The energy levels of the devices with different HTLs; (b) the *J-V* curves of devices with different HTLs (under AM 1.5G); (c) dark *J-V* scans of

devices.

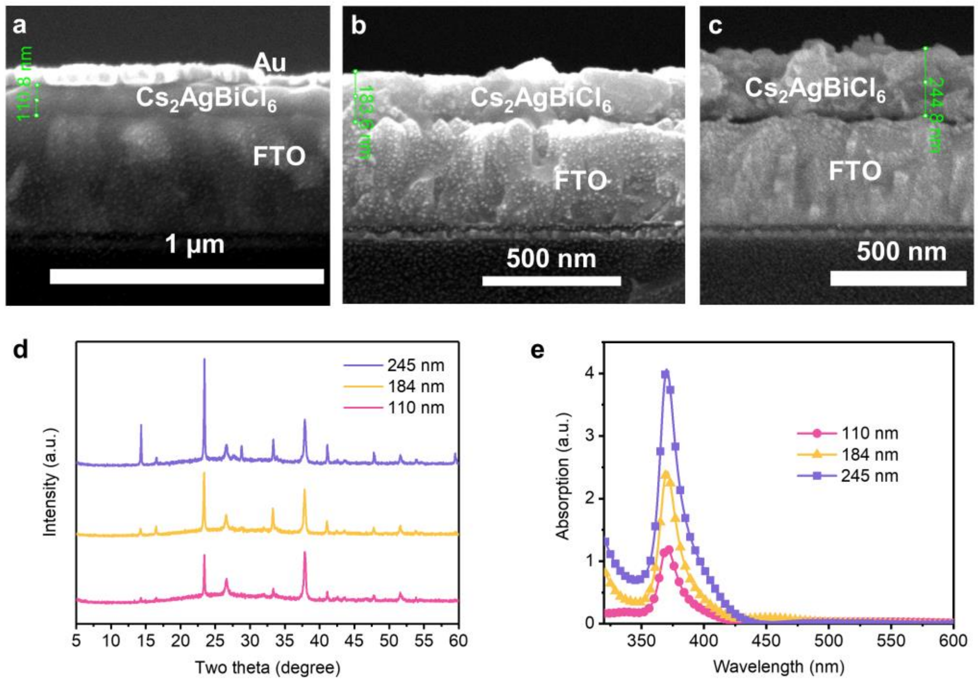

Figure S5. The cross-sectional SEM images of films with (a) 110 nm, (b) 184 nm and (c) 245 nm; (d) XRD patterns of films with different thicknesses; (e) absorption spectrum of $Cs_2AgBiCl_6$ films with different thicknesses.

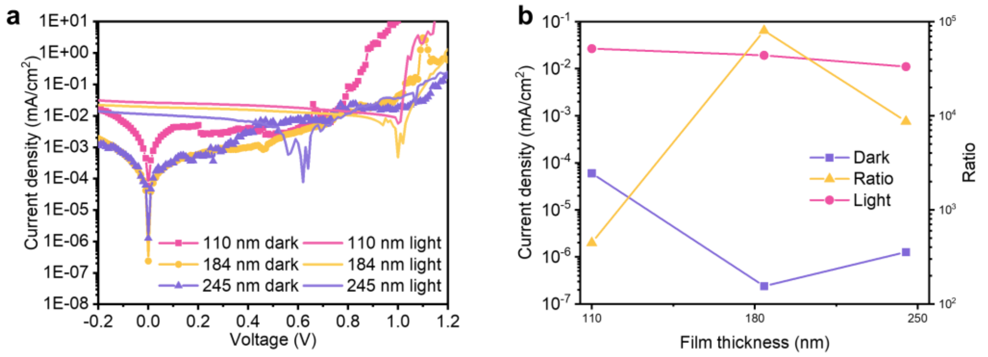

Figure S6. (a) The dark and light *J-V* curves of devices with different thickness; (b) thickness-dependent dark current density, light current density and ratio of light current density divided by dark current density under 0V (the light intensity is 1 mW/cm^2 at 365nm illumination).

Table S1. Summary of dark current density, light current density and on-off ratio of films with different thickness.

Film thickness (nm)	Dark current density (mA/cm^2)	Light current density (mA/cm^2)	Ratio of Light/dark
110	5.98E-5	0.0267	446
184	2.39E-7	0.0192	80343
245	1.27E-6	0.0111	8724

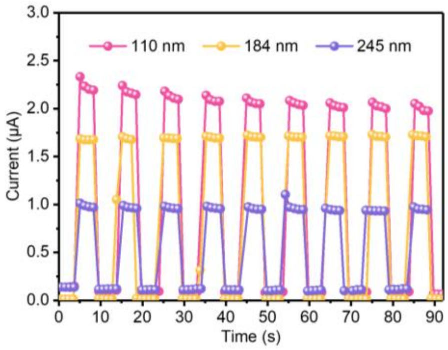

Figure S7. Continuous light response of devices with different active layer thickness.

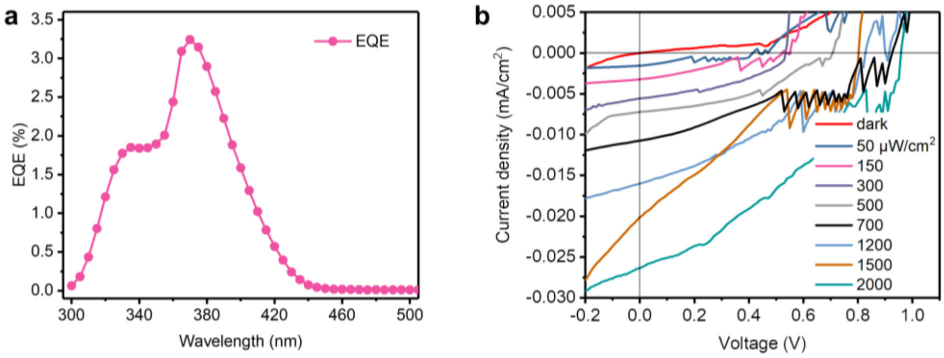

Figure S8. (a) EQE of photodetection devices; (b) *J-V* curves of devices under different light intensity which are used to power-dependent detection performance.

Table S2. Comparison of device performance between our $Cs_2AgBiCl_6$ UV PDs and literature reported UV PDs.

Photo detectors	Device type	Detection range (nm)	Dark current (nA)	Detectivity (Jones)	Toxicity	Stability	Ref.
$(PEA)_2PbBr_4$ single crystal	MSM	?-426 nm	2.20×10^{-4}	1.55×10^{13} @365 nm	Toxic	4 months	2
$MAPbCl_3$ single crystal	MSM	?-431 nm	4.15×10^{1}	1.20×10^{10} @365 nm	Toxic	12 hours reported	3
$MAPbCl_3$ film	Photodiode	About 300-400 nm	1.60	-	Toxic	Unstable (3 min)	4

GaN film	MSM	-	-	1.24×10^{9} @325 nm	A little toxic	-	5
SnO_2 microwire	Photodiode	About 275-350 nm	4.00×10^{-3}	5.41×10^{11} @315 nm	Environment-friendly	-	6
TiO_2 nanorod arrays	Photodiode	?-400 nm	-	2.50×10^{12} @380 nm	Environment-friendly	-	7
$Cs_2AgBiBr_6$ film	Photodiode	About 300-525 nm	0.95	2.10×10^{10} @350 nm	Environment-friendly	6 months	8
$Cs_2AgInCl_6$ single crystal	MSM	?-410 nm	0.01	9.60×10^{11} @365 nm	Environment-friendly	2 months	9
**$Cs_2AgBiCl_6$ film**	**Photodiode**	**300-438 nm**	**2.15×10^{-2}**	**1.11×10^{12} @365 nm**	**Environment-friendly**	**4 months**	**Our work**

Reference:

1. Yang, J.; Bao, C.; Ning, W.; Wu, B.; Ji, F.; Yan, Z.; Tao, Y.; Liu, J. M.; Sum, T. C.; Bai, S.; Wang, J.; Huang, W.; Zhang, W.; Gao, F., Stable, High‐Sensitivity and Fast‐Response Photodetectors Based on Lead‐Free Cs2AgBiBr6 Double Perovskite Films. Advanced Optical Materials 2019.

2. Zhang, Y.; Liu, Y.; Xu, Z.; Ye, H.; Li, Q.; Hu, M.; Yang, Z.; Liu, S., Two-dimensional (PEA)2PbBr4 perovskite single crystals for a high performance UV-detector. J Mater Chem C 2019.

3. Maculan, G.; Sheikh, A. D.; Abdelhady, A. L.; Saidaminov, M. I.; Haque, M. A.; Murali, B.; Alarousu, E.; Mohammed, O. F.; Wu, T.; Bakr, O. M., CH3NH3PbCl3 Single Crystals: Inverse Temperature Crystallization and Visible-Blind UV-Photodetector. *J Phys Chem Lett* **2015,** *6* (19), 3781-6.

4. Zheng, E.; Yuh, B.; Tosado, G. A.; Yu, Q., Solution-processed visible-blind UV-A photodetectors based on CH3NH3PbCl3 perovskite thin films. *J Mater Chem C* **2017,** *5* (15), 3796-3806.

5. Gundimeda, A.; Krishna, S.; Aggarwal, N.; Sharma, A.; Sharma, N. D.; Maurya, K. K.; Husale, S.; Gupta, G., Fabrication of non-polar GaN based highly responsive and fast UV photodetector. *Appl Phys Lett* **2017,** *110* (10), 103507.

6. Cai, J.; Xu, X.; Su, L.; Yang, W.; Chen, H.; Zhang, Y.; Fang, X., Self-Powered n-SnO2/p-CuZnS Core–Shell Microwire UV Photodetector with Optimized Performance. *Advanced Optical Materials* **2018,** *6* (15), 1800213.

7. Gao, Y.; Xu, J.; Shi, S.; Dong, H.; Cheng, Y.; Wei, C.; Zhang, X.; Yin, S.; Li, L., TiO2 Nanorod Arrays Based Self-Powered UV Photodetector: Heterojunction with NiO Nanoflakes and Enhanced UV Photoresponse. *Acs Appl Mater Inter* **2018,** *10* (13), 11269-11279.

8. Wu, C.; Du, B.; Luo, W.; Liu, Y.; Li, T.; Wang, D.; Guo, X.; Ting, H.; Fang, Z.; Wang, S.; Chen, Z.; Chen, Y.; Xiao, L., Highly Efficient and Stable Self-Powered Ultraviolet and Deep-Blue Photodetector Based on Cs2AgBiBr6/SnO2 Heterojunction. *Advanced Optical Materials* **2018**.

9. Luo, J.; Li, S.; Wu, H.; Zhou, Y.; Li, Y.; Liu, J.; Li, J.; Li, K.; Yi, F.; Niu, G.; Tang, J., Cs2AgInCl6 Double Perovskite Single Crystals: Parity Forbidden Transitions and Their Application For Sensitive and Fast UV Photodetectors. *ACS Photonics* **2017,** *5* (2), 398-405.
